# Gut microbiota-induced elevation of succinate exacerbates diabetic myocardial ischemia/reperfusion injury by promoting macrophage polarization

**DOI:** 10.3389/fimmu.2026.1749185

**Published:** 2026-06-10

**Authors:** Yang Wu, Juan Dou, Min Liu, Song Peng, Wenyuan Li

**Affiliations:** 1 Department of Anesthesiology, Renmin Hospital of Wuhan University, Wuhan, Hubei, China; 2 Sterilization and supply center, Renmin Hospital of Wuhan University, Wuhan, Hubei, China; 3 Department of Cardiology, The Central Hospital of Wuhan, Huazhong University of Science and Technology, Wuhan, Hubei, China

**Keywords:** diabetes, gut microbiota, macrophage, myocardial ischemia/reperfusion injury, succinate

## Abstract

**Introduction:**

Patients with diabetes exhibit increased susceptibility to myocardial ischemia/reperfusion (IR) injury. Emerging evidence highlights gut microbiota-derived microbial metabolites as critical modulators of the gut–heart axis. This study examined the pathological role of succinate, a crucial metabolite derived from the microbiota, in the progression of diabetic myocardial IR injury.

**Methods:**

The gut microbiota composition and succinate levels in diabetic mice were analyzed. Changes in succinate levels were subsequently assessed following the depletion of the gut microbiota induced by antibiotics. Diabetic mice with genetic ablation of Sucnr1 were administered exogenous succinate supplementation prior to IR induction. In vitro, conditioned medium transfer systems were utilized to coculture macrophages with cardiomyocytes subjected to hypoxia/reoxygenation injury, and the extent of cardiomyocyte damage was evaluated.

**Results:**

Cardiac succinate accumulation during diabetes progression is linked to gut microbial dysbiosis, which is characterized by an imbalance between succinate-producing and succinate-consuming bacteria. Antibiotic-mediated depletion of the gut microbiota reversed this succinate accumulation, demonstrating that microbial communities constitute a major source of cardiac succinate. Furthermore, succinate drove the polarization of cardiac macrophages and exacerbated diabetes-associated myocardial IR injury. Conversely, genetic knockdown of Sucnr1 in both in vitro and in vivo models ameliorated the detrimental effects of succinate.

**Discussion:**

Cardiac succinate accumulation occurs during diabetes progression and is associated with specific gut microbial dysbiosis, which is characterized by a disrupted equilibrium between succinate-producing and succinate-consuming bacteria. This pathological accumulation of succinate exacerbates diabetes-associated myocardial IR injury by driving macrophage polarization through activation of the SUCNR1 signaling pathway.

## 1 Introduction

Diabetes represents a major health issue worldwide, and it is estimated that by the year 2030, the worldwide prevalence of diabetes will increase to an estimated 552 million individuals across developed and developing nations (1, 2). Cardiovascular disease (CVD) is the leading cause of morbidity and mortality in individuals with diabetes (3). Notably, type 2 diabetes (T2D) increases susceptibility to myocardial ischemia/reperfusion (IR) injury, culminating in severe left ventricular (LV) dysfunction following revascularization procedures (4, 5). Despite substantial progress in diagnostic and therapeutic strategies that have notably increased early survival rates following myocardial infarction, patients with diabetes continue to exhibit persistently elevated mortality (6). Consequently, mitigating the susceptibility of the diabetic myocardium to reperfusion injury represents a critical need in clinical cardiology.

Accumulating evidence indicates that immune dysregulation plays a central role in the pathophysiology of myocardial IR injury (7). Among the infiltrating immune cells, macrophages represent the predominant population and exhibit significant functional plasticity. During myocardial IR injury, macrophages constitute the predominant infiltrating immune cell population and display marked functional plasticity (8). This plasticity is functionally critical as macrophages polarize into distinct functional phenotypes that dichotomously regulate postinjury outcomes by modulating both inflammatory cascades and reparative pathways (9).Proinflammatory macrophages (M1 phenotype) infiltration injured tissue during the early phase, followed by the recruitment of anti-inflammatory macrophages (M2 phenotype). Ischemia initiates M1 macrophage polarization, a process characterized by increased expression of proinflammatory cytokines, which partially exacerbates IR injury (10, 11). The immune response plays a pivotal role in the pathophysiology of IR injury, in which attenuation of M1 polarization is correlated with reduced myocardial damage and diminished cardiomyocyte apoptosis (12). One promising approach towards this objective may involve regulating the population of cardiac macrophages.

In addition to inflammatory mediators, recent studies have highlighted the crucial role of metabolic signals in the regulation of macrophage function (13). Emerging evidence indicates that the gut microbiota produces a diverse array of metabolites, many of which enter the systemic circulation and exert bioactive effects (14). Microbial metabolites, such as short-chain fatty acids (SCFAs), function as pivotal signaling molecules that modulate immune homeostasis not only within the gut but also in remote organs, including the heart. Dysbiosis of the gut microbiota and consequent alterations in microbial metabolites have been strongly linked to CVD and its major risk factors, including T2D. Among these metabolites, succinate has been identified as a critical immunometabolic signaling molecule. Studies have demonstrated that both ischemia and diabetes can further exacerbate succinate accumulation, thereby positioning succinate as a master regulator of metabolic disturbance and ischemic pathology.

The identification of the G protein-coupled receptor 91 (GPR91), also known as succinate receptor 1 (SUCNR1), has provided a mechanistic basis for succinate-mediated immune regulation (15). SUCNR1 is highly expressed on macrophages and acts as a specific sensor for extracellular succinate. Accumulating evidence demonstrates that succinate signaling through SUCNR1 drives divergent responses in macrophages, with the succinate-SUCNR1 axis playing a crucial role in regulating macrophage polarization and function (15, 16).

However, the specific function of succinate in influencing macrophage activity in diabetic hearts, especially during myocardial IR injury, is remains poorly understood. Therefore, targeting microbial succinate production may represent a promising therapeutic strategy (17). We hypothesize that succinate overproduction in diabetes is attributable to specific gut microbiota dysbiosis. This pathological accumulation of succinate drives proinflammatory macrophage polarization via SUCNR1 activation in the diabetic myocardium, thereby exacerbating diabetes-associated myocardial IR injury.

## 2 Materials and methods

### 2.1 Ethics statement and animal model

All animal procedures were approved by the Animal Ethics Committee of Renmin Hospital, Wuhan University, and conducted in accordance with the Guide for the Care and Use of Laboratory Animals (8th ed., 2011) issued by the U.S. National Institutes of Health (NIH). Male C57BL/6J mice (7 weeks old) were purchased from Beijing Vital River Laboratory Animal Technology (Beijing, China). Additionally, Sucnr1^-/-^ mice and their wild-type (WT) littermates, obtained from heterozygous breeding pairs, were acquired from Cyagen Biosciences, Inc. (Suzhou, China). The mice were housed in a specific pathogen-free (SPF) barrier facility at a temperature range of 22–25 °C under a 12-hour light/dark cycle. They were acclimated to the SPF setting for one week before any experiments were conducted. During the adaptation phase, the mice were cycled through different cages before randomization into specific treatment groups. Animal care and husbandry practices adhered to established guidelines, and the allocation of animals to experimental groups was randomized. To eliminate potential cage-related influences, two mice were individually housed in sterile isolation cages (18).

### 2.2 Induction of diabetes

**Figure 1:**
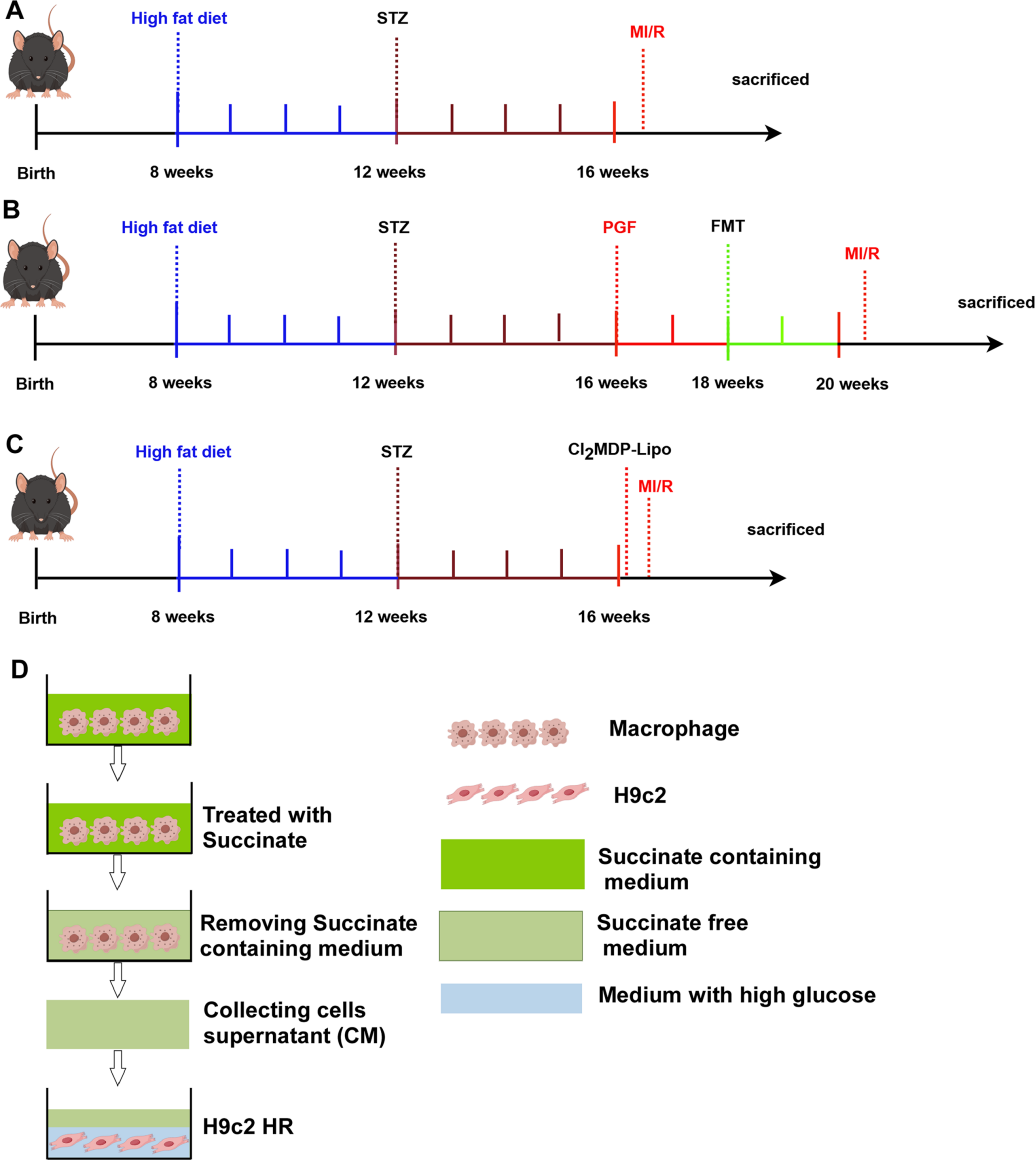
Schematic of the animal and cell experimental protocols. **(A)** A T2D mouse model was induced by a HFD and low-dose STZ injection, followed by myocardial IR. **(B)** Diabetic PGF mice were established by ABX and underwent myocardial IR operation. **(C)** To deplete macrophages, diabetic mice received an intraperitoneal injection of Cl2MDP-Lipo 3 days prior to myocardial IR injury. **(D)** Protocol of the CM transfer model. IR, ischemia/reperfusion; T2D, type 2 diabetes; HFD, high-fat diet; ABX: antibiotic mixture; PGF: pseudo germ-free; STZ, streptozocin; HR: hypoxia reoxygenation; CM: Conditioned medium; HG: high glucose.

T2D was induced in mice via a 4-week high-fat diet (HFD) feeding protocol, followed by a single intraperitoneal injection of low-dose streptozocin (STZ) (90 mg/kg, dissolved in 0.1 M sodium citrate buffer, pH 4.5), which is consistent with established methods (19). Mice were diagnosed with diabetes if fasting blood glucose levels exceeded 11.1 mmol/L (measured after a 12-hour fast) at 4 weeks post-STZ injection (**Figure 1A**). Age-matched control mice were maintained on a normal caloric diet (ND) and administered vehicle control (0.01 M sodium citrate buffer).

### 2.3 Establishment of a pseudo germ-free (PGF) mouse model

PGF mouse models were established prior to transplantation and are widely utilized in fecal microbiota transplantation (FMT) studies (20). A 7-day high-dose antibiotic mixture (ABX: 100 mg/kg metronidazole, 100 mg/kg neomycin, 50 mg/kg vancomycin, and 1 mg/mL ampicillin) was suspended in saline administered by oral gavage (21, 22). The experimental timeline is illustrated in **Figure 1B**.

### 2.4 Faecal microbiota transplantation

Faecal samples obtained from diabetic mice were homogenized in sterile saline at a concentration of 0.1 g/ml. A 0.2 mL aliquot of the solution was administered daily via oral gavage to PGF diabetic mice (FMT group) for 14 consecutive days (23) (**Figure 1B**). All the mice were maintained on ad libitum food and water, and the gavage procedures were performed under aseptic conditions. Following the 14-day FMT protocol, the mice underwent myocardial IR injury induction via 30 min of left anterior descending (LAD) coronary artery occlusion followed by 24 h of reperfusion. Post-IR, cardiac tissues and serum samples were collected for further analyses.

### 2.5 Depletion of macrophages

To deplete macrophages within the diabetic myocardium, mice were intraperitoneally injected with dichloro-methylene diphosphonate liposomes (Cl_2_MDP-Lipo) (Liposoma, C-005) 3 days before myocardial IR surgery, as described in a previous study (24)(**Figure 1C**). The control group of mice received an equivalent volume of liposome-encapsulated PBS (Veh).

### 2.6 Myocardial IR surgery

Myocardial IR surgery was conducted following previously established procedures (19). The mice were anaesthetized via an intraperitoneal injection of sodium pentobarbital (100 mg/kg), followed by endotracheal intubation and mechanical ventilation using a Harvard Apparatus rodent ventilator. Myocardial IR injury was induced by performing a left thoracotomy to exteriorize the heart, followed by ligation of the LAD coronary artery with an 8–0 silk suture slipknot. After 30 min of ischemia, the slipknot was released to initiate reperfusion for 24 h. The control group underwent identical surgical procedures, including suture placement beneath the LAD, without ligation (6). After 24 h of reperfusion, the mice were subjected to deep anesthesia before being euthanized via rapid excision of the heart. Perioperative analgesia was provided by subcutaneous administration of buprenorphine (0.05mg/kg).

### 2.7 Determination of cardiac function and measurement of infarct size

After 24 h of reperfusion, cardiac function was assessed via echocardiography. In the parasternal LV long-axis view, measurements were taken for the LV internal dimension at systole and the LV internal dimension at diastole. Computer algorithms were used to calculate the left ventricular ejection fraction (LVEF) and left ventricular shortening fraction (LVFS). After completion of functional determination, the myocardial infarct size was determined via an Evans blue and triphenyl tetrazolium chloride (TTC) double-staining protocol as previously described (24). The area at risk (AAR) was defined as the Evans blue-unstained region, and the infarct area (IF) as the TTC-negative zone. The myocardial infarct size was calculated as the ratio of the infarct area to the area at risk (IF/AAR).

### 2.8 Histological analysis and tissue immunofluorescence

After reperfusion, cardiac tissues were fixed in 4% paraformaldehyde, paraffin-embedded, and sectioned into 5 µm slices. For immunofluorescence staining, paraffin-embedded sections were deparaffinized in xylene, followed by heat-induced antigen retrieval. Nonspecific binding was blocked with 5% horse serum in PBS for 30 min at room temperature. The sections were incubated overnight at 4 °C with primary antibodies and subsequently incubated with species-matched fluorescent secondary antibodies. Digital images were acquired using an Olympus BX53 fluorescence microscope.

### 2.9 Cell culture and intervention

The murine macrophage RAW 264.7 cell line (ATCC TIB-71) was cultured in modified Eagle’s medium (DMEM) supplemented with 10% foetal bovine serum (FBS). To achieve Sucnr1 knockdown in RAW 264.7 macrophage, RAW 264.7 cells were transfected with short hairpin RNA (shRNA) constructs via Lipo8000™ transfection reagent (Beyotime, Shanghai, China). Following transfection, the medium was replaced with complete DMEM, and subsequent experiments were conducted 24 hours post transfection. H9c2 cells (Cell Bank of the Chinese Academy of Sciences, Shanghai, China) were maintained in modified DMEM supplemented with 10% (v/v) FBS at 37 °C in a 5% CO_2_ incubator. To mimic diabetic conditions *in vitro*, H9c2 cells were exposed to serum-free DMEM under high glucose (HG) conditions, containing 25 mM glucose (19). For the hypoxia reoxygenation (HR) injury model, the cells were subjected to hypoxia (94% N_2_+5% CO_2_+1% O_2_) for 4 h, followed by 2 h of reoxygenation (95% air+5% CO_2_) under normoxic conditions (6, 25).

### 2.10 Conditioned medium (CM) transfer systems and cell experiment grouping

As illustrated in **Figure 1D**, a CM transfer system was established based on established methodologies to elucidate the mechanistic role of succinate in exacerbating HR-induced injury in H9c2 cardiomyocytes via M1 macrophage polarization (26). Briefly, RAW 264.7 macrophages were seeded and exposed to succinate at concentrations of 0, 0.1, 0.5, 1, or 5 mM for 24 h to establish dose-dependent relationship. The mRNA expression levels of M1 polarization markers (*Tnf, Il1b, Il6, and Nos2*) were then measured to determine the optimal succinate concentration for subsequent experiments. To specifically assess the contribution of macrophage-derived secretory factors, the succinate-containing medium was then replaced with fresh, succinate−free medium, and cells were cultured for an additional 24 h. The resulting CM was collected, diluted 1:3 with DMEM, and applied to H9c2 cells subjected to HR injury. To establish a direct causal link between SUCNR1 activation and cardiomyocyte injury, we performed loss-of-function experiments by transducing RAW 264.7 macrophages with lentiviral shRNA targeting *Sucnr1* (sh*Sucnr1*).

The experimental groups were defined as follows: HR+HG group: H9c2 cells cultured in high-glucose (25 mM) serum-free DMEM under HR conditions; HR+HG+CM (Control) group: H9c2 cells cultured in HG DMEM under HR with CM from unstimulated control macrophages; HR+HG+CM group: H9c2 cells cultured in HG DMEM under HR with CM collected from succinate-stimulated RAW 264.7 macrophages; HR+HG+CM+sh*Sucnr1* group: H9c2 cells cultured in HG DMEM under HR with CM from sh*Sucnr1-*transfected RAW 264.7 macrophages stimulated with succinate.

### 2.11 Lactate dehydrogenase (LDH) activity, troponin T (cTnT), and cell viability

Following reperfusion or experimental treatment, blood samples or culture medium supernatants were immediately harvested from the heart or cultured cardiomyocytes. These samples were then centrifuged at 8,000×g for 5 min at 4 °C. The cleared supernatants were aliquoted and stored at −80 °C until LDH or cTnT levels were quantified. Cardiomyocyte viability was quantified according to the manufacturer’s protocol for the Cell Counting Kit-8 (CCK-8; DOJINDO, Japan) (25).

### 2.12 Flow cytometry analysis (FCM)

Noncardiomyocytes were isolated from mouse hearts and subjected to myocardial tissue cytometry analysis, as described in a previous study (27). Briefly, under deep anesthesia, the heart was exposed and the right ventricle was immediately flushed with 7 mL of EDTA buffer to clear blood. The heart was then excised and placed into a 60-mm Petri dish containing fresh EDTA buffer. Cardiac digestion was performed by sequential injection of 10 mL EDTA buffer, 3 mL perfusion buffer, and 30–50 mL collagenase-based digestion buffer into the LV while the ascending aorta was clamped with Reynolds forceps to force perfusion through the coronary vasculature. The digested myocardium was gently teased into 1 mm³ pieces and triturated to obtain a single-cell suspension, which was then passed through a 100-μm nylon mesh. To enrich nonmyocyte populations, the cell suspension was subjected to four sequential rounds of gravity settling (20 min each) in 15-mL conical tubes. After each round, the supernatant containing nonmyocytes was carefully collected and pooled, while the pellet was progressively enriched in cardiomyocytes. The pooled nonmyocyte supernatants were centrifuged at 300 × g for 5 min at 4 °C to pellet the cells. The pellet was resuspended in PBS and layered onto a discontinuous Percoll gradient consisting of 40% and 70% Percoll (prepared by diluting stock isotonic Percoll [Cytiva, 17089102] with PBS). The gradient was centrifuged at 500 × g for 30 min at 4 °C with slow acceleration and no brake. Mononuclear cells, including macrophages, were collected from the interface between the 40% and 70% Percoll layers, washed twice with PBS, and counted. Cells were preincubated with an Fc receptor blocking solution (BD Biosciences, Cat# 553141) for 10 min at 4 °C to reduce nonspecific antibody binding. They were then stained with fluorophore-conjugated primary antibodies or appropriate isotype-matched controls for 30 min at 4 °C in the dark. After washing, cells were resuspended in staining buffer and analyzed using a CytoFLEX flow cytometer. Data were processed with FlowJo software (v10.8.1, BD Biosciences).

### 2.13 TUNEL staining assay to evaluate the degree of cardiomyocyte apoptosis

Myocardial apoptosis was evaluated using the terminal deoxynucleotidyl transferase-mediated dUTP nick-end labelling (TUNEL) assay, which uses the TUNEL Cell Death Detection Kit (Roche, USA). The apoptosis index (AI) was calculated by dividing the number of TUNEL-positive nuclei by the total number of nuclei using ImageJ software (6).

### 2.14 Western blot analysis

Western blot analysis was performed in accordance with established protocols (25, 28). Briefly, total protein was extracted from homogenized tissues or cultured cells via ice-cold cell lysis buffer supplemented with protease and phosphatase inhibitors. The protein lysates were separated using 10% SDS–PAGE and subsequently transferred electrophoretic ally to polyvinylidene fluoride membranes. The membranes were blocked with 5% nonfat milk in Tris-buffered saline containing 0.1% Tween-20 for 1 h at room temperature. This was followed by an overnight incubation at 4 °C with primary antibodies diluted in blocking buffer. After washing, the membranes were incubated with species-matched horseradish peroxidase (HRP)-conjugated secondary antibodies for 1 h at room temperature. The bands were detected using the Odyssey CLx Imaging System and quantified as normalized band intensity ratios relative to β-actin or GAPDH (loading controls). The following antibodies were used: SUCNR1 (Novus Biologicals, #NBP1-00861, 1:1,000), AKT (Cell Signaling Technology, #4691, 1:1,000), phospho-AKT (Ser473; Cell Signaling Technology, #4060, 1:1,000), and hypoxia-inducible factor-1α (HIF-1α) (Santa Cruz Biotechnology, #sc-53546, 1:1,000).

### 2.15 Real-time polymerase chain reaction

Reverse transcription–quantitative PCR (RT–qPCR) was conducted as previously described (28). In summary, total RNA was reverse-transcribed into complementary DNA (cDNA) using the PrimeScript RT Reagent Kit (ServiceBio, Cat# G3337) in accordance with the manufacturer’s instructions. The primer pairs, detailed in **Supplementary Table 1**, were designed using NCBI Primer-BLAST, and amplicon specificity was verified through melt curve analysis. Gene expression levels were normalized to those of β-actin and determined using the 2−ΔΔCt method.

### 2.16 16S rRNA gene amplicon sequencing microbe analysis

Faecal samples were aseptically collected into sterile 2 mL cryovials, rapidly frozen on dry ice and stored at −80 °C until microbial genomic analysis was conducted. Total genomic DNA was extracted from homogenized faecal aliquots using the QIAamp Fast DNA Stool Mini Kit (QIAGEN, Cat# 51604) according to the manufacturer’s instructions, and the DNA concentration was quantified via NanoDrop One spectrophotometry (Thermo Fisher Scientific). The hypervariable V3–V4 regions of the bacterial 16S rRNA genes were amplified on an Illumina MiSeq platform (2 × 250 bp paired-end reads; Illumina) by Personalbio Co., Ltd. (Shanghai, China). The raw FASTQ files were processed using QIIME2 v2023.2, which included demultiplexing, quality filtering (Phred score ≥30), and denoising with DADA2. Operational taxonomic units (OTUs) were clustered at 97% sequence similarity via VSEARCH. The Chao, Shannon, and Simpson indices were employed to assess the alpha diversity of the intestinal microbiome. Principal Coordinate Analysis (PCoA) was utilized to examine the beta diversity of the gut flora (29).

### 2.17 Statistical analysis

All data are expressed as the mean ± standard error of the mean (SEM). Statistical analyses were conducted utilizing either the t-test or one-way/two-way analysis of variance (ANOVA) tests. These analyses were executed using Prism 9 software (GraphPad, San Diego, CA, USA), with differences considered statistically significant at a *P* value of less than 0.05. Furthermore, β-diversity, reflecting the variation in microbial composition between groups, was assessed using the Bray-Curtis distance and visualized through PCoA plots generated with the R language package.

## 3 Results

### 3.1 Elevated succinate levels in diabetic mice are associated with changes in the gut microbiota composition

**Figure 2:**
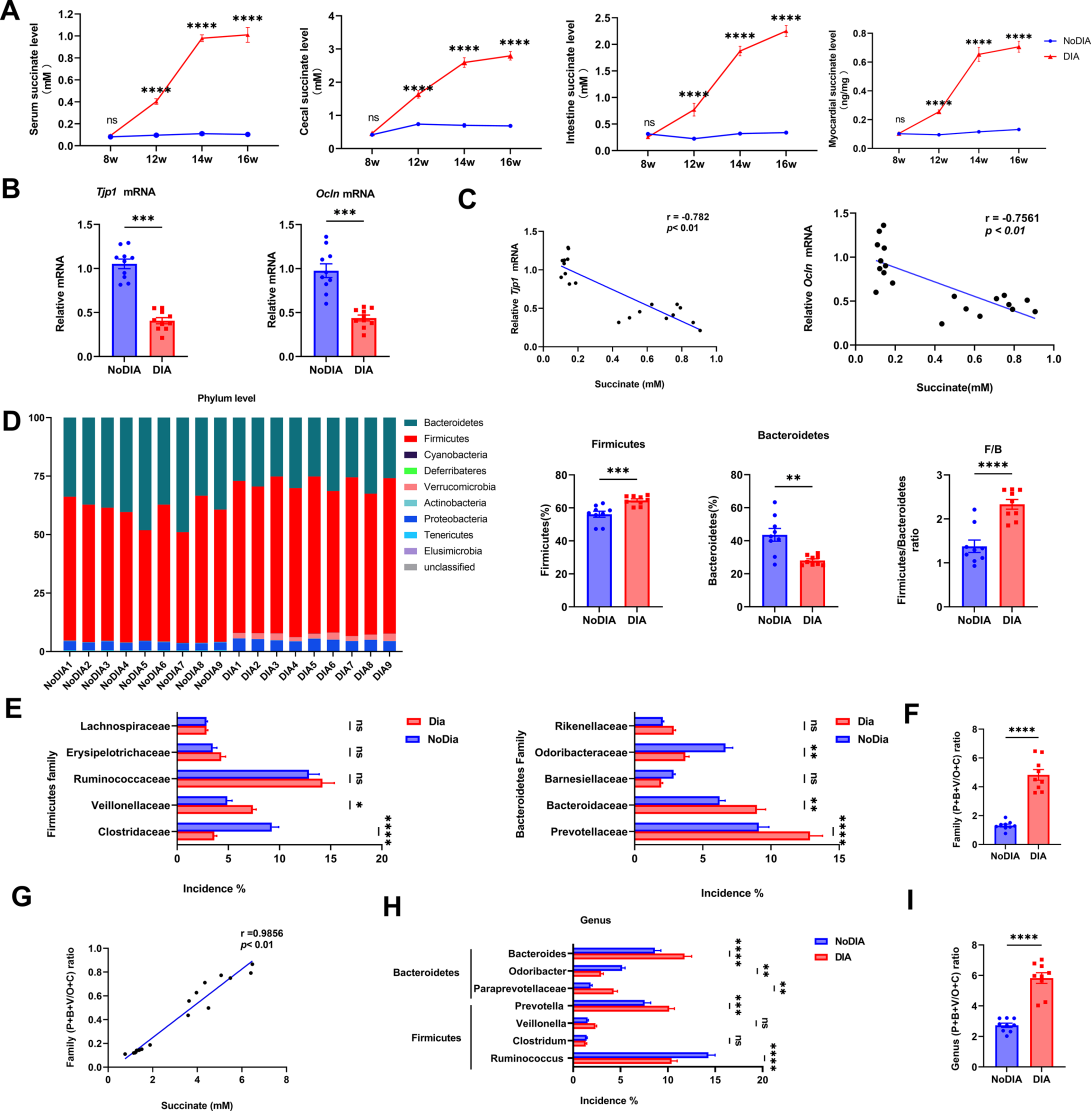
Succinate levels in the diabetic myocardium are elevated during diabetes and are associated with alterations in specific gut microbiota. **(A)** Succinate levels in the serum, caecal contents, intestine, and myocardium after diabetes induction (n = 6). **(B)** mRNA expression of the tight junction genes *Tjp1* and *Ocln* (n = 6). **(C)** Negative correlation between myocardial succinate levels and the mRNA expression of *Tjp1* and *Ocln* (n =10). **(D)** Altered microbiota composition at the phylum level and an increased Firmicutes/Bacteroidetes (F/B) ratio after diabetes induction (n = 9). **(E)** Increased relative abundance of succinate-producing bacterial families (Prevotellaceae, Bacteroidaceae, and Veillonellaceae) and decreased abundance of succinate-consuming families (Odoribacteraceae and Clostridaceae) in diabetic mice (n = 9). **(F)** The ratio of succinate-producing to succinate-consuming bacteria (P+B+V/O+C) at the family level (n = 9). **(G)** The ratio of succinate producers to consumers was positively correlated with myocardial succinate levels (n = 9). **(H)** Comparison of the relative abundance of succinate-producing and succinate-consuming bacteria at the genus level between diabetic and nondiabetic mice (n = 9). **(I)** The ratio of P+B+V/O+C at the genus level (n = 9). Data are expressed as mean ± standard error of the mean. ^*^P < 0.05, ^**^P < 0.01, ^***^P < 0.001, ^****^P < 0.0001, ns, not significant. F/B: Firmicutes/Bacteroidetes; P+B+V/O+C: Prevotellaceae + Bacteroidaceae + Veillonellaceae/Odoribacteraceae + Clostridaceae; IR, ischemia/reperfusion; NoDIA: nodiabetes, DIA:diabetes.

Succinate concentrations were quantified at various time points in diabetic mice to assess changes in relation to the duration of diabetes. Compared with their nondiabetic counterparts, diabetic mice presented a progressive increase in succinate levels, with significant accumulation observed in the intestine, caecal contents, circulating serum, and myocardial tissue (**Figure 2A**).

Tight junction proteins, such as ZO-1 and occludin, are essential for maintaining the structural and functional integrity of the intestinal epithelial barrier. The mRNA expression levels of *Tjp1* and *Ocln* were found to be reduced in the intestines of diabetic mice compared to nondiabetic mice (**Figure 2B**). Western blot analysis revealed a significant decrease in the protein expression levels ofZO-1 and occludin in the diabetic group (**Supplementary Figure 1**). Additionally, a strong inverse correlation was observed between myocardial succinate levels and the mRNA expression of *Tjp1* and *Ocln* at the transcriptional level (**Figure 2C**). These findings indicate that succinate accumulation in diabetic cardiac tissue may be closely linked to increased intestinal permeability.

The analysis of the gut microbiota revealed that diabetes significantly impacted the Chao, Shannon, and Simpson indices, which reflect reduced microbial diversity, richness, and biodiversity at the phylum level (**Supplementary Figure 2**). Diabetic mice exhibited an increased Firmicutes/Bacteroidetes (F/B) ratio, indicating a shift in the composition of the gut microbiota (**Figure 2D**). As shown **Figure 2E**, this alteration was characterized by an increased relative abundance of succinate-producing bacterial families, including Prevotellaceae, Bacteroidaceae and Veillonellaceae, compared with nondiabetic mice. In contrast, succinate-consuming families such as Odoribacteraceae and Clostridaceae were less abundant in diabetic mice. The ratio of succinate producers to consumers (Prevotellaceae + Bacteroidaceae + Veillonellaceae/Odoribacteraceae + Clostridaceae) (P+B+V/O+C) was significantly greater in diabetic mice than in nondiabetic mice (**Figure 2F**) and was positively correlated with myocardial succinate levels (**Figure 2G**). At the genus level, diabetic mice presented enrichment of succinate-producing taxa (Prevotella and Bacteroides) alongside a significant reduction in succinate-consuming genera (Ruminococcus and Odoribacter) (**Figure 2H**). The P+B+V/O+C ratio at the genus level was also significantly greater in diabetic mice (**Figure 2I**).

### 3.2 Increase in succinate levels induced by the gut microbiota exacerbates myocardial IR injury in diabetes

**Figure 3:**
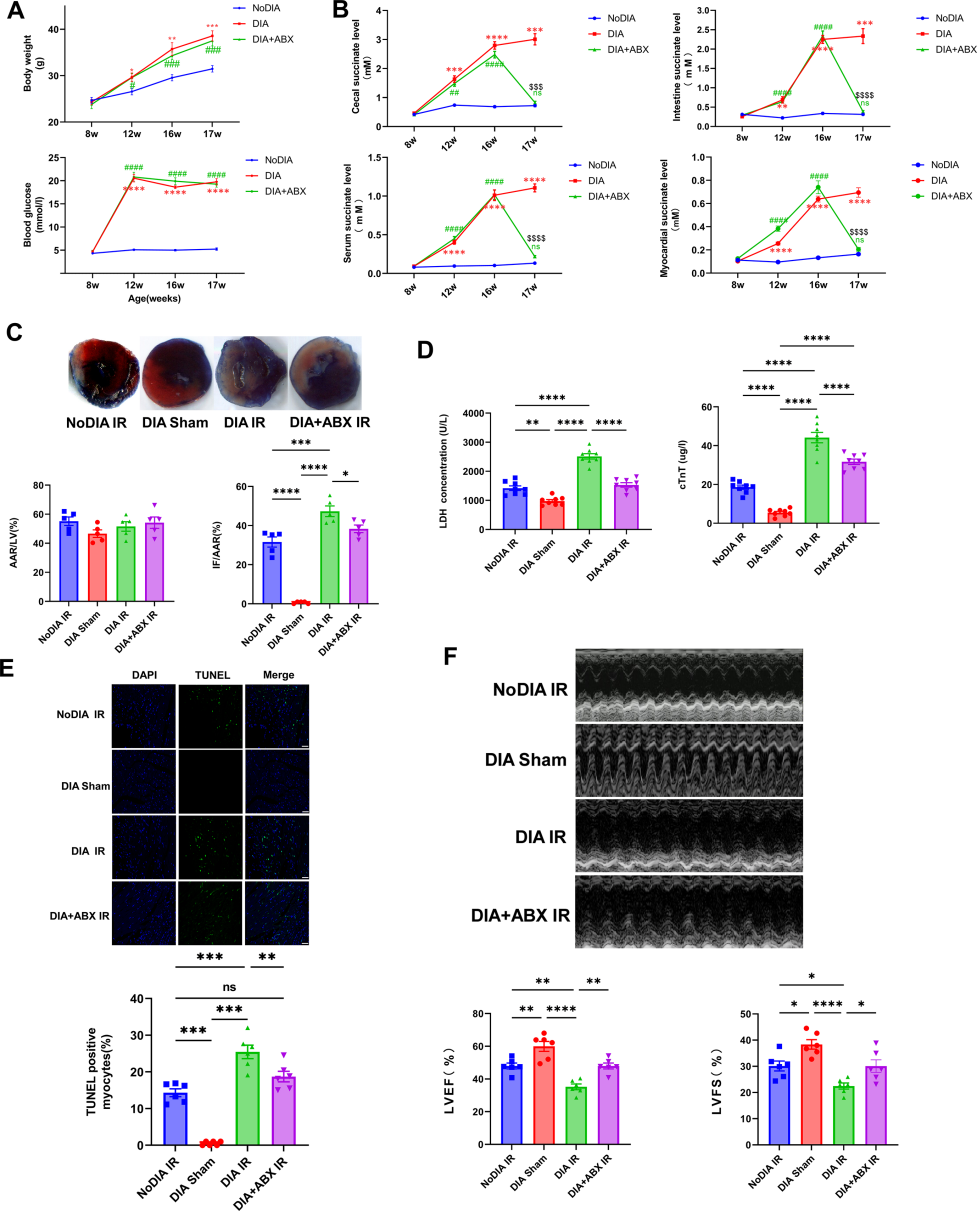
Depletion of the gut microbiota reverses elevated succinate levels and attenuates diabetes-aggravated myocardial IR injury. **(A)** Body weight and blood glucose levels were monitored after STZ or ABX administration (n=10). **(B)** Succinate levels in the cecal contents, intestine, serum, and myocardium after ABX treatment (n = 6). **(C)** Myocardial infarct size assessed by Evans blue/TTC double staining (n = 5). **(D)** Serum LDH and cTnT levels in diabetic and nodiabetic mice post-IR (n=8). **(E)** Cardiomyocyte apoptosis determined by TUNEL assay. Scale bar = 50 μm (n=6). **(F)** Representative images and quantification of LVEF and LVFS (n=6). Data are expressed as mean ± standard error of the mean. ^*^P < 0.05, ^**^P < 0.01, ^***^P < 0.001, ^****^P < 0.0001, ^#^P < 0.05, ^##^P < 0.01, ^####^P < 0.0001, ^$$$^P < 0.001, ^$$$$^P < 0.0001, ns, not significant. IR, ischemia/reperfusion; NoDIA: nodiabetes, DIA:diabetes, ABX: antibiotic mixture; LVEF: left ventricular ejection fraction; LVFS: left ventricular shortening fraction.

After demonstrating elevated succinate levels and gut microbiota dysbiosis in diabetic mice, we are now testing the causal role of the microbiota via antibiotic depletion. Broad-spectrum ABX was administered orally to diabetic mice with the aim of depleting their gut microbiota. After high-dose ABX treatment, a PGF diabetic mouse model was established, as evidenced by the significant differences in α-diversity indices compared with the untreated diabetic group (**Supplementary Figures 3A–C**). Compared with non-ABX-treated diabetic mice, PGF diabetic mice presented no significant changes in body weight or blood glucose levels (**Figure 3A**). Notably, ABX-treated diabetic mice presented marked reductions in succinate concentrations across the intestines, caecal contents, serum, and myocardial tissues (**Figure 3B**). Additionally, ABX treatment resulted in a smaller myocardial infarction size, reduced LDH and cTnT levels, and fewer apoptotic cells (**Figures 3C–E**). Echocardiography was performed one day after myocardial IR injury to assess cardiac function. Echocardiographic analysis revealed that ABX treatment ameliorated myocardial IR injury, as evidenced by a significant reduction in LVEF and LVFS (**Figure 3F**). This finding underscores the critical role of succinate in exacerbating myocardial IR injuries in diabetes.

### 3.3 Effects of gut microbiota transplantation on diabetic myocardial IR injury

**Figure 4:**
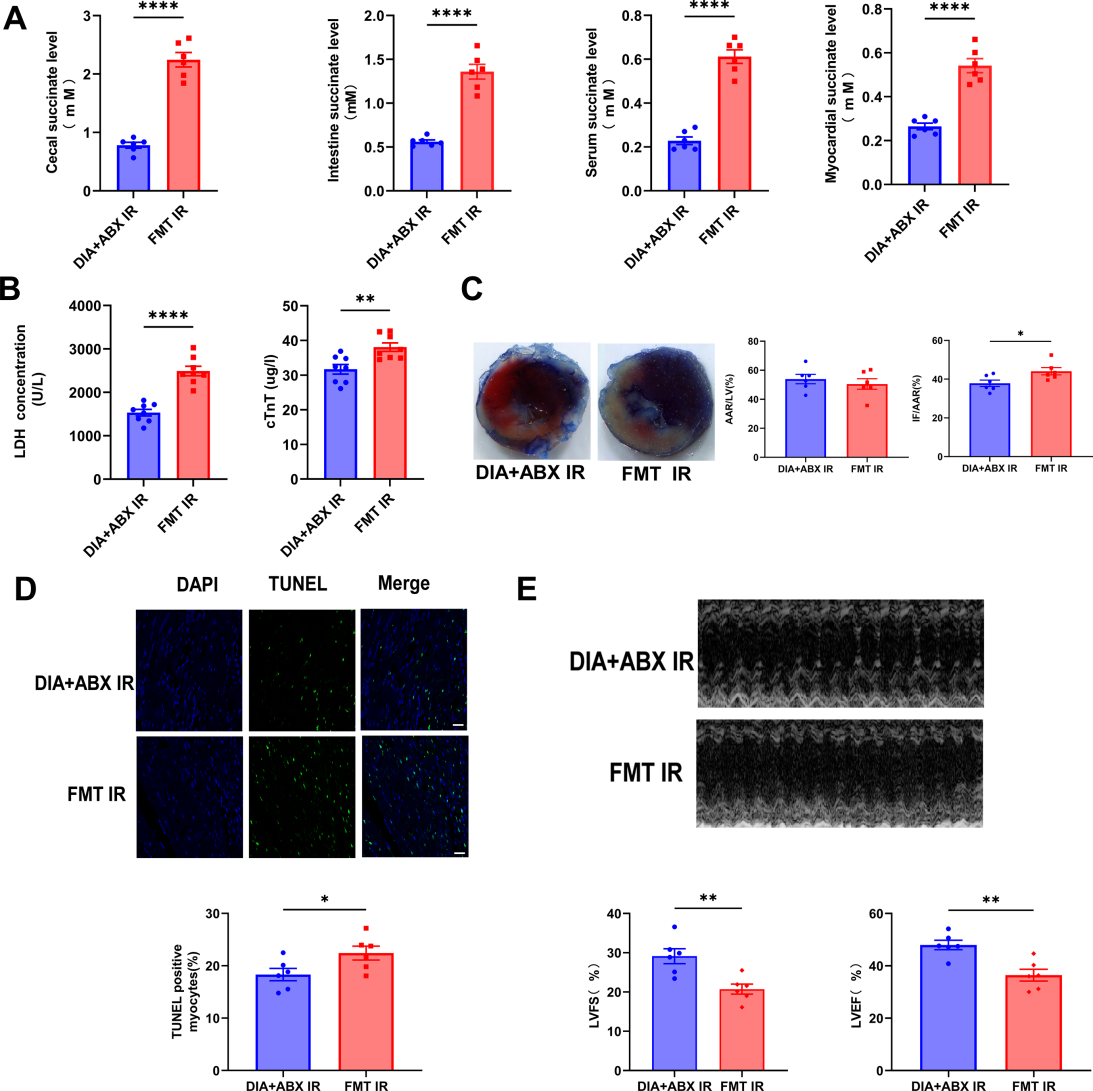
FMT from diabetic mice increases succinate levels and exacerbates myocardial IR injury in diabetic PGF mice. **(A)** Succinate levels in the cecal contents, intestine, serum and myocardium, (n=6). **(B)** Serum LDH and cTnT levels post-IR (n=8). **(C)** Myocardial infarct size assessed by Evans blue/TTC double staining (n = 5). **(D)** Cardiomyocyte apoptosis was assessed by a TUNEL assay. Scale bar = 50 μm. (n=6). **(E)** Representative images and quantification of LVEF and LVFS (n=6). Data are expressed as mean ± standard error of the mean. ^*^ P < 0.05, ^**^ P < 0.01, ^***^P < 0.001, ^****^P < 0.0001. FMT: Faecal microbiota transplantation; IR, ischemia/reperfusion; NoDIA: nodiabetes, DIA:diabetes, ABX: antibiotic mixture; LVEF: left ventricular ejection fraction; LVFS: left ventricular shortening fraction.

To further verify that the gut microbiota is responsible for increased succinate levels and aggravated myocardial IR injury in diabetes, we performed FMT experiments. Compared with the ABX-treated diabetic group, a significant difference in microbiota compositions was observed in the diabetic mice after FMT (**Supplementary Figure 3D**). The FMT group presented elevated succinate levels in the caecal contents, intestines, serum, and myocardial tissues (**Figure 4A**). Furthermore, following myocardial IR injury, the FMT group presented increased LDH and cTnT levels, a larger myocardial infarct size, and a greater number of apoptotic cells (**Figures 4B–D**). Echocardiographic analysis revealed that diabetic gut microbiota transplantation exacerbated IR injury, as evidenced by significant impairments in LVEF and LVFS (**Figure 4E**). These findings confirm that succinate is a critical pathophysiological mediator linking gut microbiota activity to the progression of diabetic myocardial IR injury.

### 3.4 Succinate induced increased polarization of M1 macrophages and aggravated myocardial IR injury in diabetic mice

**Figure 5:**
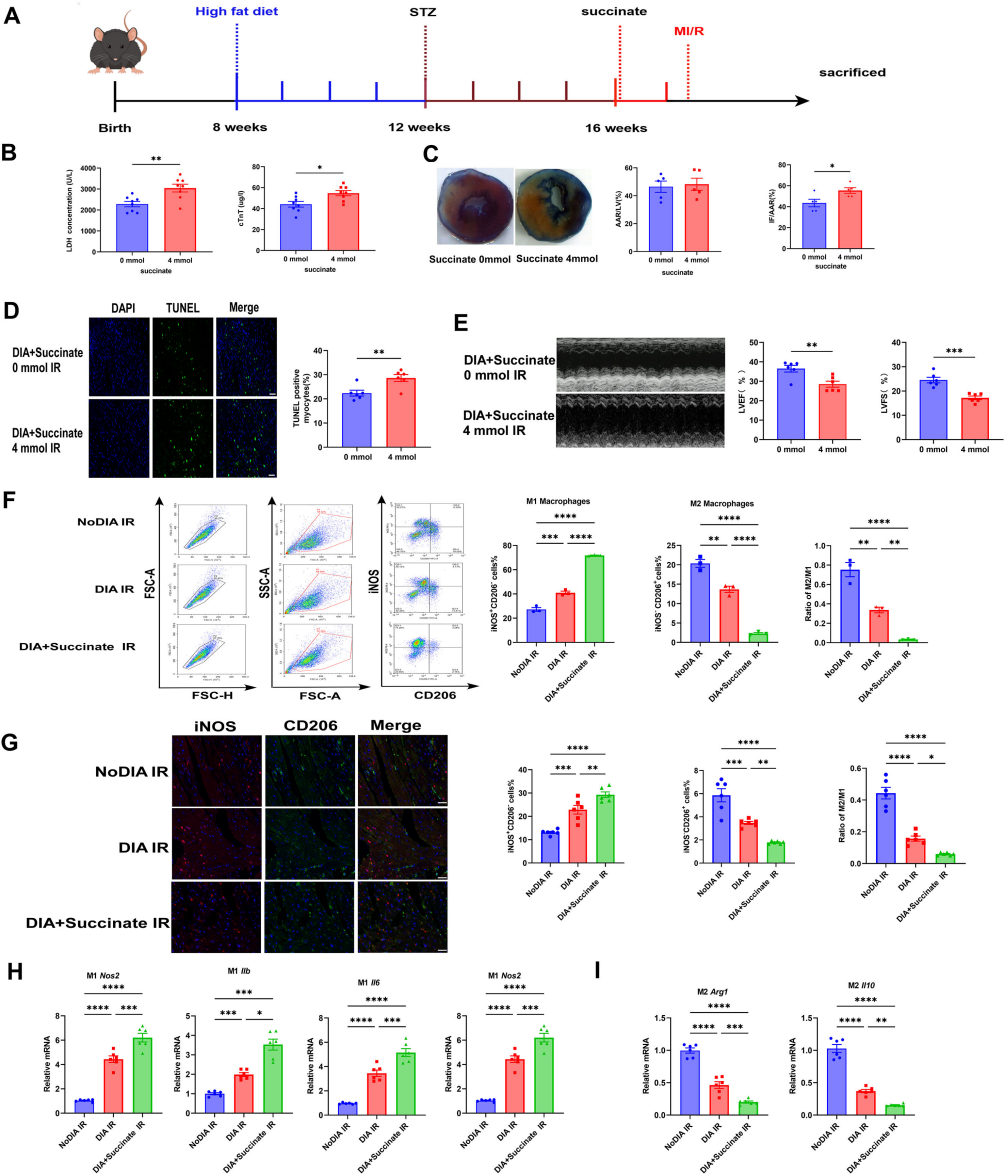
Succinate exacerbates diabetic myocardial IR injury and promotes macrophage polarization towards the M1 phenotype. **(A)** Experimental protocol: diabetic mice received daily intraperitoneal injection of succinate for 7 days before myocardial IR injury. **(B)** Serum LDH and cTnT levels in diabetic and nodiabetic mice post-IR (n=8). **(C)** Myocardial infarct size assessed by Evans blue/TTC double staining (n = 5). **(D)** Cardiomyocyte apoptosis determined by TUNEL assay. Scale bar = 50 μm (n=6). **(E)** Representative images and quantification of LVEF and LVFS (n=6). **(F)** Representative flow cytometry plots showing iNOS^+^CD206^-^ (M1) and iNOS^-^CD206^+^ (M2) macrophages (n=3). **(G)** quantitative analysis of M1 macrophages (iNOS^+^CD206^-^) and M2 macrophages (iNOS^-^CD206^+^) in the myocardium (n = 3). **(H)** mRNA expression of M1-related genes (*Tnf, Il1, Il6, and Nos2*) in cardiac tissue (n = 6). **(I)** mRNA expression of M2-related genes (Arg1 and *Il10*) in cardiac tissue (n = 6). Data are expressed as mean ± standard error of the mean. ^*^ P < 0.05, ^**^P < 0.01, ^***^P < 0.001, ^****^P < 0.0001. IR, ischemia/reperfusion; NoDIA: nodiabetes, DIA:diabetes; LVEF: left ventricular ejection fraction; LVFS: left ventricular shortening fraction.

After confirming that succinate exacerbates myocardial IR injury in diabetic mice, we next examined the role of macrophages in mediating this effect. To investigate the pathological role of succinate in macrophage polarization *in vivo*, diabetic mice received daily intraperitoneal injections of succinate (4 mmol/kg/day) or vehicle prior to myocardial IR injury (**Figure 5A**) (30). Compared with vehicle (succinate, 0 mmol/kg/day), succinate significantly increased the LDH and cTnT levels, myocardial infarct size, and apoptosis (**Figures 5B–D**). Furthermore, succinate administration exacerbated cardiac dysfunction, as evidenced by a significant decrease in LVEF and LVFS (**Figure 5E**). FCM analysis following IR injury revealed marked polarization towards proinflammatory M1-like macrophage phenotypes in diabetic mice, alongside a significantly lower M2/M1 ratio than in nondiabetic group (**Figure 5F**). Notably, succinate administration increased M1 macrophage infiltration while suppressing M2 polarization, further decreasing the M2/M1 ratio. Immunohistochemical analysis of myocardial tissue in the infarct area revealed that succinate treatment significantly increased M1 macrophage infiltration and reduced the M2/M1 polarization ratio (**Figure 5G**). Consistently, succinate administration promoted significant M1 macrophage polarization, as evidenced by upregulated expression of proinflammatory markers (*Tnf, Il1b, Il6, and Nos2*) and downregulated expression of M2-related markers (*Arg1* and *Il10*) (**Figures 5H, I**).

### 3.5 Macrophage involvement in the effect of succinate exacerbates diabetic myocardial IR injury

**Figure 6:**
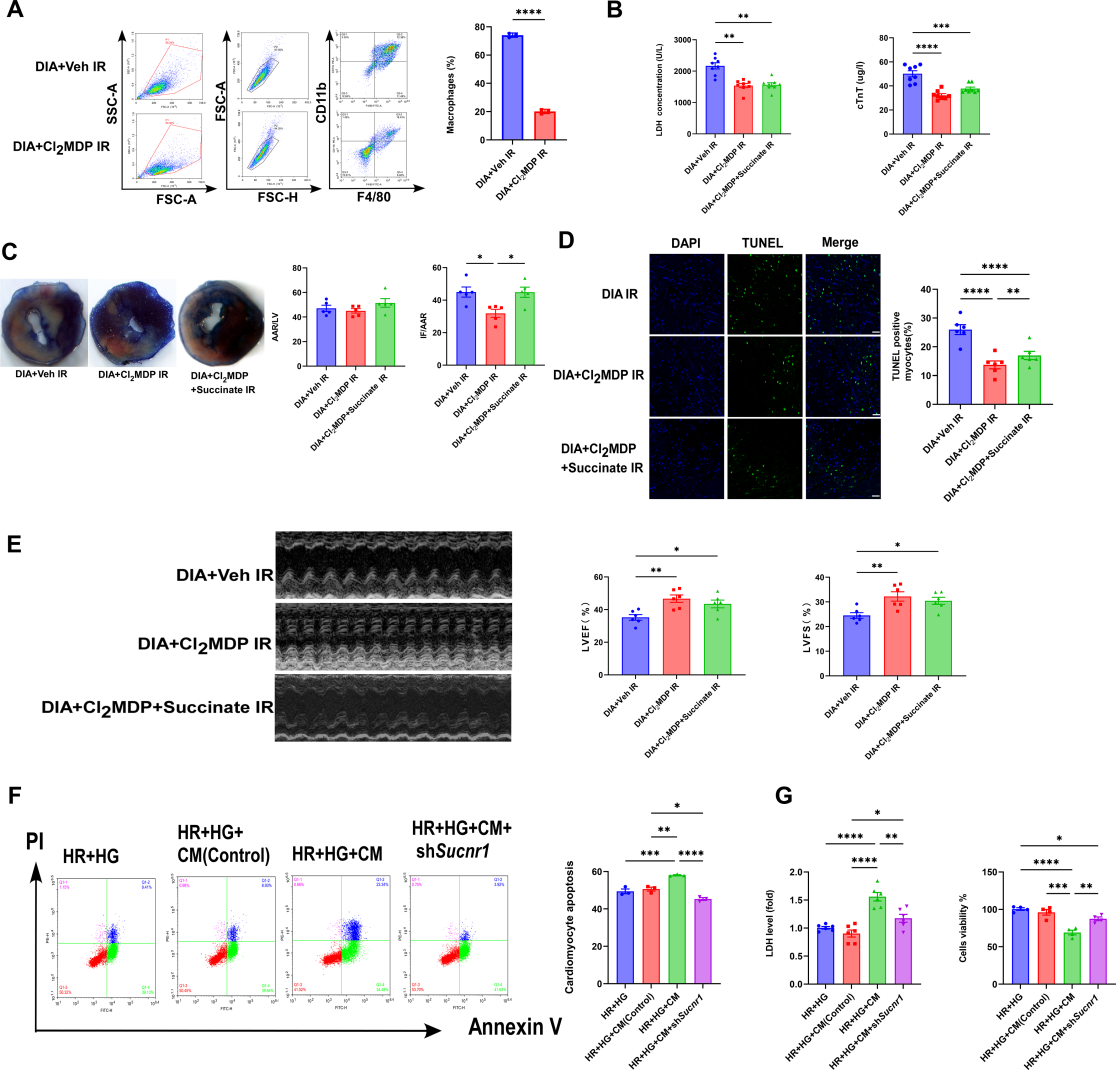
Macrophage activation is essential for succinate-mediated diabetic myocardial IR injury. **(A)** Flow cytometry analysis of cardiac macrophages (F4/80^+^CD11b^+^) post myocardial IR (n=3). **(B)** Serum LDH and cTnT levels post-IR (n=8). **(C)** Myocardial infarct size assessed by Evans blue/TTC double staining (n = 5). **(D)** Cardiomyocyte apoptosis determined by TUNEL assay. Scale bar = 50 μm (n=6). **(E)** Representative images and quantification of LVEF and LVFS (n=6). **(F)** Representative flow cytometry plots of H9c2 cardiomyocyte apoptosis in the indicated groups (n = 3). **(G)** LDH release and cell viability of H9c2 cells in the indicated groups (n = 6). Data are expressed as mean ± standard error of the mean. ^*^P < 0.05, ^**^P < 0.01, ^***^P < 0.001, ^****^P < 0.0001. IR: ischemia/reperfusion; NoDIA: nodiabetes; DIA: diabetes; Veh: Vehicle. HR: hypoxia reoxygenation; CM: Conditioned medium; HG: high glucose. Group definitions: HR+HG group: H9c2 cells cultured in high-glucose (25 mM) serum-free DMEM under HR conditions; HR+HG+CM (Control) group: H9c2 cells cultured in HG DMEM under HR with CM from unstimulated control macrophages; HR+HG+CM group: H9c2 cells cultured in HG DMEM under HR with CM collected from succinate-stimulated (0.5 mM) RAW 264.7 macrophages; HR+HG+CM+sh*Sucnr1* group: H9c2 cells cultured in HG DMEM under HR with CM from sh*Sucnr1* transfected RAW 264.7 macrophages stimulated with succinate.

To further confirm the critical role of macrophages, a systemic depletion approach was used in mice through the intraperitoneal injection of Cl_2_MDP-Lipo (24, 31). FCM analysis revealed a marked reduction in cardiac macrophage populations following Cl_2_MDP-Lipo treatment (**Figure 6A**). Additionally, Cl_2_MDP-Lipo administration attenuated myocardial IR injury in diabetic mice, as evidenced by reduced LDH and cTnT levels, a smaller infarct size, and fewer TUNEL-positive apoptotic cells (**Figures 6B–D**). Moreover, diabetic mice treated with Cl_2_MDP-Lipo presented improved LVEF and LVFS (**Figure 6E**). Notably, Cl_2_MDP-Lipo treatment effectively attenuated the succinate-induced increase in myocardial infarct size and the associated cardiac dysfunction. Collectively, these findings confirm that macrophage depletion mitigates succinate-exacerbated myocardial IR injury, underscoring the critical role of macrophages in diabetic myocardial IR injury.

The next step was to assess whether succinate affects cardiomyocytes through macrophages *in vitro*. In the CM transfer model, succinate exposure induced dose-dependent upregulation of M1 polarization markers (*Tnf, Il1b, Il6, and Nos2*) in RAW 264.7 macrophages (**Supplementary Figure 4**). At 0.5 mM, all four markers exhibited robust and statistically significant upregulation compared to the PBS control. Based on the dose-response analysis, a concentration of 0.5 mM succinate was selected for all subsequent experiments to examine its direct effect on macrophage polarization. As shown in **Figures 6F and 6G**, sh*Sucnr1* significantly reduced cardiomyocyte apoptosis, decreased LDH release, and improved cell viability compared to the HR+HG+CM group. These findings indicate that succinate exacerbates HR-induced cardiomyocyte injury through macrophage-mediated mechanisms, a conclusion that is consistent with our *in vivo* observations.

### 3.6 Succinate promotes macrophage polarization through SUCNR1 and exacerbates IR injury in the diabetic myocardium

**Figure 7:**
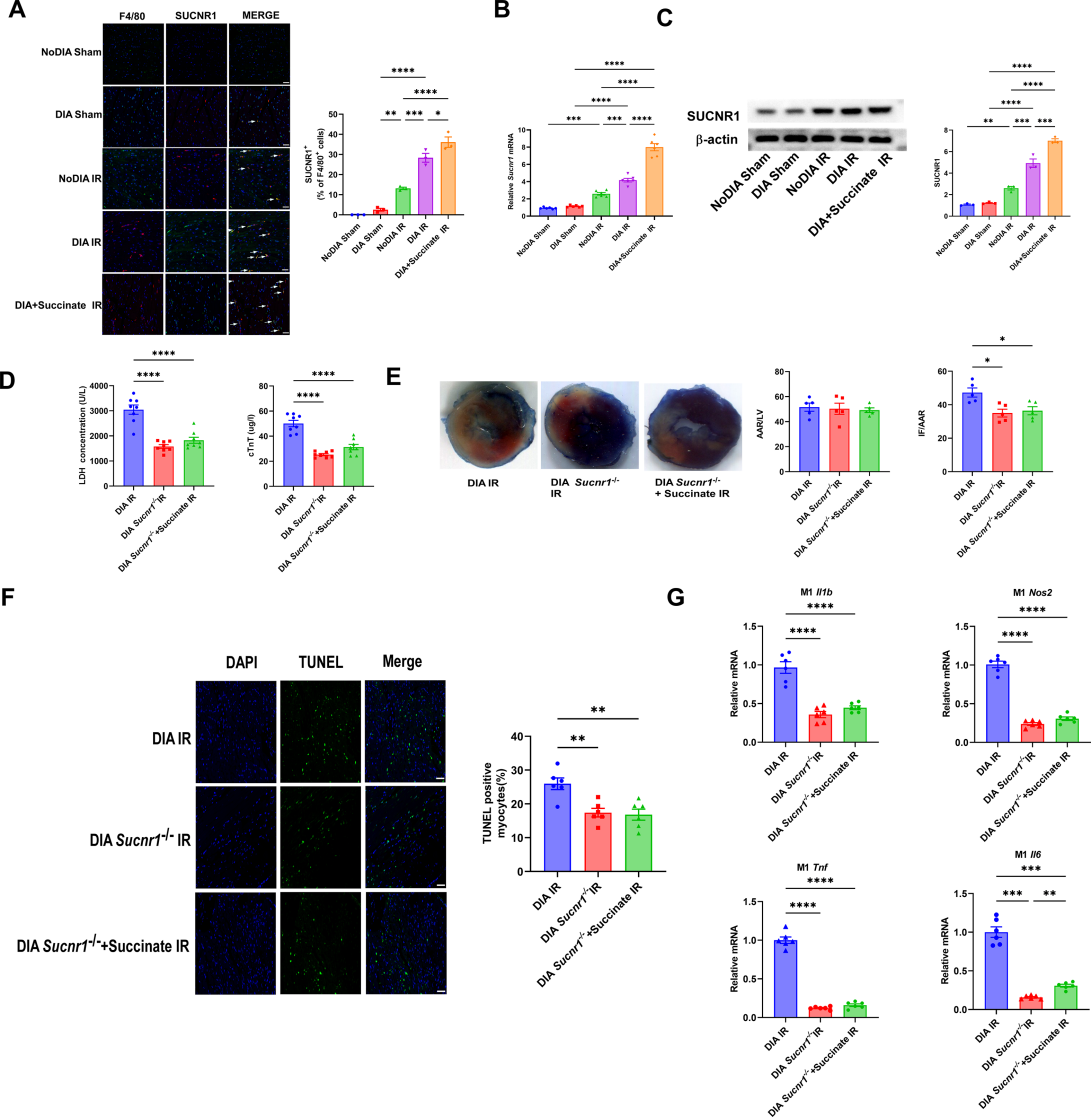
Succinate-induced macrophage polarization aggravated diabetic myocardial IR injury via SUCNR1. **(A)** Representative immunofluorescence images showing SUCNR1 and F4/80 colocalization in the infarct zone at 24 h post-I/R. Scale bar=50 µm, (n=3). **(B)** Sucnr1 mRNA levels in cardiac tissue lysates collected from the infarct zone at 24 h post-I/R (n=6). **(C)** Western blot analysis of SUCNR1 protein levels in the infarct zone at 24 h post-I/R (n=3). **(D)** Serum LDH and cTnT levels post-IR (n=8). **(E)** Myocardial infarct size assessed by Evans blue/TTC double staining (n = 5). **(F)** Cardiomyocyte apoptosis determined by TUNEL assay. Scale bar = 50 μm (n=6). **(G)** mRNA expression of M1-related genes in whole cardiac tissue collected from the infarct zone at 24 h post-I/R. (n = 6). Data are expressed as mean ± standard error of the mean. ^*^P < 0.05, ^**^P < 0.01, ^***^P < 0.001, ^****^P < 0.0001. IR, ischemia/reperfusion; NoDIA: nodiabetes, DIA:diabetes.

To determine whether SUCNR1 mediates the pro-inflammatory effects of succinate on cardiac macrophages *in vivo*, we first examined the expression pattern of SUCNR1 in the infarcted myocardium. Immunofluorescence analysis of the infarct area at 24 h post-I/R revealed that the proportion of SUCNR1^+^ cells co-expressing the macrophage marker F4/80 was significantly greater in diabetic hearts than in nondiabetic controls (**Figure 7A**). Exogenous succinate administration further promoted the recruitment of SUCNR1^+^F4/80^+^ cells to the infarct zone, with a concurrent upregulation of SUCNR1 at both the mRNA and protein levels (**Figures 7B, C**).

We next investigated whether genetic ablation of *Sucnr1* could protect against succinate-exacerbated myocardial IR injury. *Sucnr1*^-/-^ mice underwent the same HFD+STZ protocol to induce type 2 diabetes. Before surgery, no significant differences in body weight or fasting blood glucose were observed between diabetic *Sucnr1*^-/-^ and diabetic WT mice (**Supplementary Figure 5**), ruling out baseline metabolic differences as a confounding factor. Compared with diabetic WT mice, diabetic *Sucnr1*^-/-^ mice exhibited significantly attenuated myocardial IR injury, as evidenced by lower serum LDH and cTnT levels, reduced infarct size, and fewer TUNEL-positive cardiomyocytes (**Figures 7D–F**). This protection was accompanied by the downregulation of M1 macrophage polarization markers in the infarcted myocardium (**Figure 7G**), indicating that SUCNR1 is required for succinate-driven M1 polarization and the consequent exacerbation of IR injury.

### 3.7 Succinate regulates macrophage polarization in diabetic myocardial IR injury through the PI3K/AKT pathway

**Figure 8:**
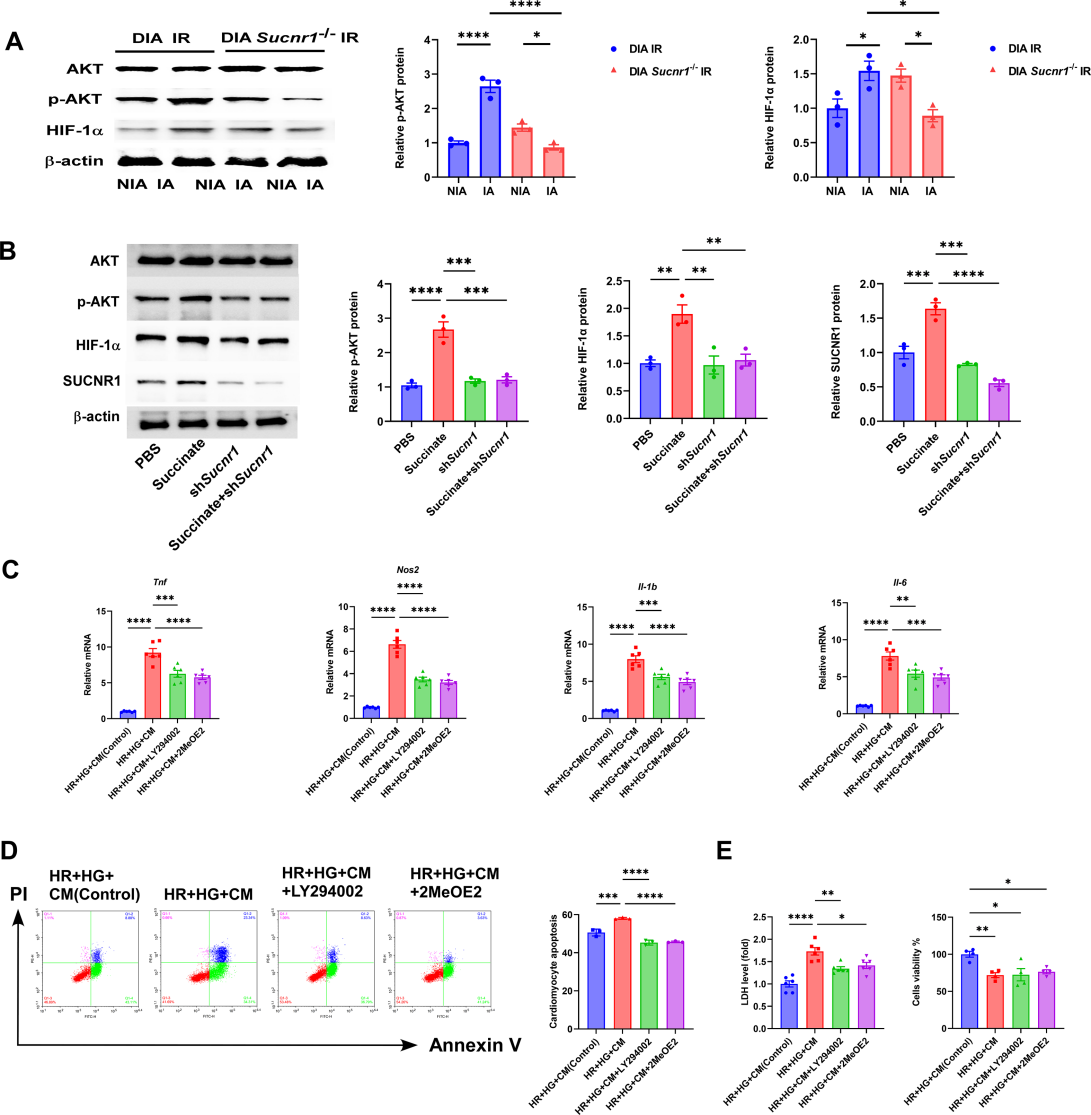
The succinate–SUCNR1 axis is required for macrophage polarization and exacerbates diabetic myocardial IR injury. **(A)** Western blot analysis of p-AKT and HIF-1α in the infarcted area (IA) and non-infarcted area (NIA) of the left ventricle collected at 24 h post-I/R from diabetic WT and Sucnr1^-^/^-^ mice. Succinate treatment increased HIF-1α and p-AKT levels, whereas these effects were reversed in Sucnr1^−/−^ diabetic mice (n=3). **(B)** Western blot analysis of p-AKT and HIF-1α in cultured RAW 264.7 macrophages. The upregulation of p-AKT and HIF-1α induced by succinate was abolished in shSucnr1-transfected macrophages (n=3). **(C)** mRNA expression of M1-related genes in RAW 264.7 macrophages treated with the HIF-1α inhibitor 2MeOE2 or the PI3K inhibitor LY294002 (n=6). **(D)** Flow cytometry analysis of H9c2 cardiomyocyte apoptosis in the indicated groups (n=6). **(E)** LDH release and cell viability of H9c2 cells in the indicated groups (n = 6). Data are expressed as mean ± standard error of the mean. ^*^P < 0.05, ^**^P < 0.01, ^***^P < 0.001, ^****^P < 0.0001. IR, ischemia/reperfusion; NoDIA: nodiabetes, DIA:diabetes, CM: Conditioned medium, HG: high glucose, HR: hypoxia reoxygenation. NIA: non-infarcted area; IA: infarcted area. Group definitions: HR+HG group: H9c2 cells cultured in high-glucose (25 mM) serum-free DMEM under HR conditions; HR+HG+CM (Control) group: H9c2 cells cultured in HG DMEM under HR with CM from unstimulated control macrophages; HR+HG+CM group: H9c2 cells cultured in HG DMEM under HR with CM collected from succinate-stimulated (0.5 mM) RAW 264.7 macrophages.

Succinate influences macrophage polarization by activating the PI3K/HIF-1α signaling pathway through SUCNR1 (32), and the functions of the associated molecules have been examined. Western blot analysis was performed using protein lysates from the infarcted area (IA) and the remote non-infarcted area (NIA) of the LV collected at 24 h post-IR from diabetic WT and Sucnr*1*^-/-^ mice. An increase in AKT phosphorylation and HIF-1α levels was observed in the IA compare to NIA at 24 h after reperfusion in diabetic group, which was consistent with increased macrophage activation within the infarcted area (**Figure 8A**). Notably, this response was abolished in diabetic *Sucnr1*^-/-^ mice. In parallel with the *in vitro* experiments, succinate exposure increased AKT phosphorylation and HIF-1α levels in RAW 264.7 macrophages at 24 h, and these effects were markedly attenuated by sh*Sucnr1* (**Figure 8B**).

To further investigate the involvement of the PI3K/AKT pathway in succinate-driven macrophage polarization, RAW 264.7 macrophages were treated with the PI3K inhibitor LY294002 or the HIF-1α inhibitor 2MeOE2. Both inhibitors significantly attenuated the succinate-induced upregulation of M1 polarization markers (**Figure 8C**). In the CM transfer model, pharmacological inhibition of PI3K or HIF-1α abrogated succinate-mediated macrophage polarization and subsequent apoptosis of H9c2 cardiomyocytes (**Figure 8D**). The results were consistent with the findings from the LDH and cell viability assays (**Figure 8E**).

## 4 Discussion

**Figure 9:**
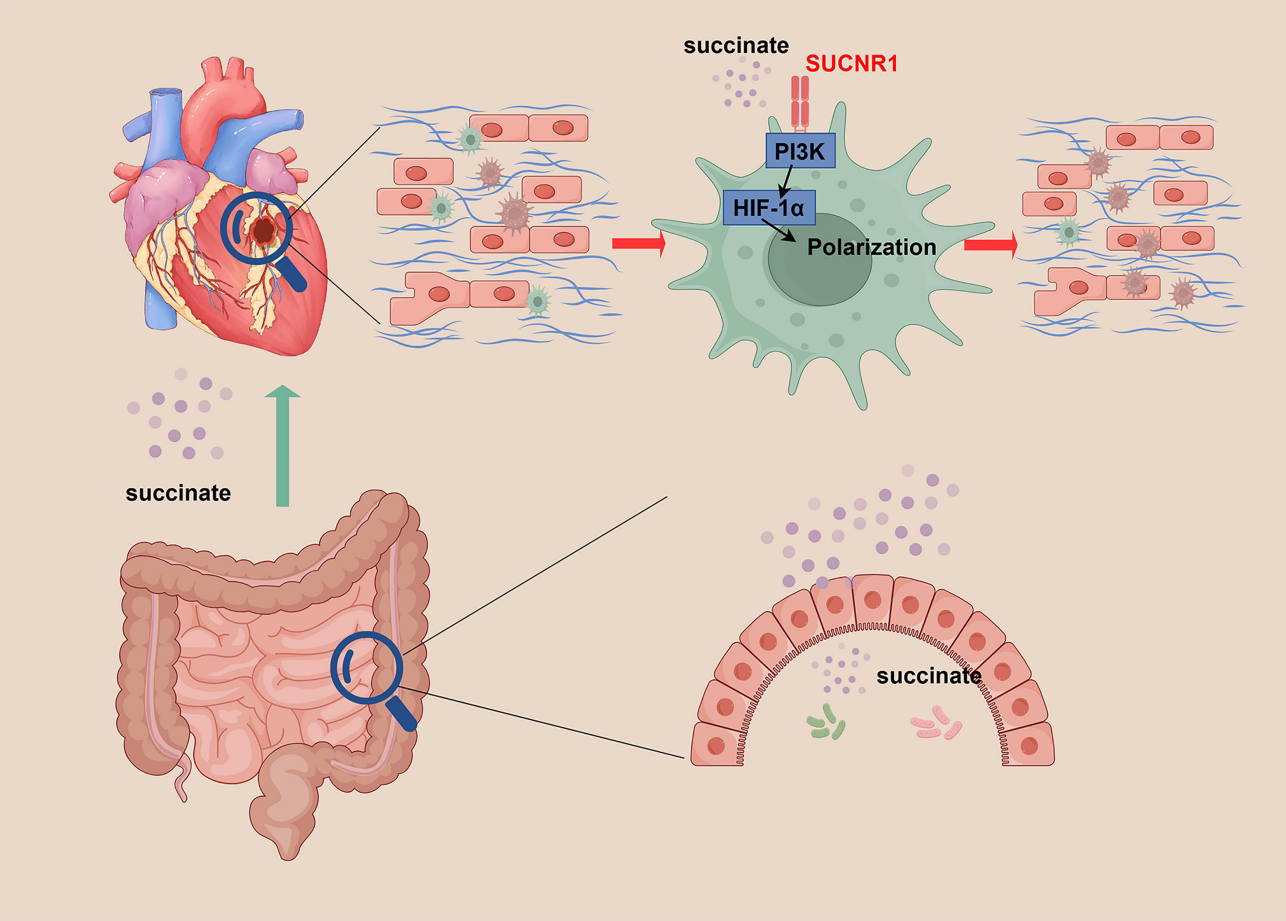
Gut microbiota-derived succinate exacerbates diabetic myocardial ischemia/reperfusion (IR) injury by promoting M1 macrophage polarization via the SUCNR1–PI3K/AKT–HIF-1α pathway.

This study identifies a novel gut microbiota–succinate–macrophage–cardiomyocyte axis as a pathogenic mechanism linking diabetes to exacerbated myocardial IR injury. We demonstrate that diabetes-induced gut microbial dysbiosis—characterized by an imbalance between succinate-producing bacteria (Prevotellaceae, Bacteroidaceae, Veillonellaceae) and succinate-consuming bacteria (Odoribacteraceae, Clostridaceae)—drives abnormal succinate accumulation in the circulation and myocardium. This gut microbiota-derived succinate promotes cardiac macrophage M1 polarization via SUCNR1-dependent activation of the PI3K/AKT–HIF-1α signaling pathway, ultimately exacerbating myocardial IR injury. These findings highlight the therapeutic potential of targeting the succinate–SUCNR1 axis in diabetic cardiovascular complications (**Figure 9**).

Gut microbiota-derived metabolites have emerged as promising clinical biomarkers and mechanistic mediators in cardiovascular disease (33, 34). Among these, succinate has attracted considerable attention as both a TCA cycle intermediate and an extracellular signaling molecule (35). Circulating succinate levels are significantly elevated in type 2 diabetes and ischemic heart disease (36, 37), yet the pathological significance of this elevation in the context of diabetic myocardial IR injury has remained unclear. Our study extends these clinical observations by providing mechanistic evidence that elevated succinate is not merely a passive biomarker, but an active driver of cardiac injury in the diabetic state.

Previous studies identified Prevotellaceae and Veillonellaceae as primary succinate producers, whereas Odoribacteraceae and Clostridaceae predominantly mediate succinate consumption (36). The (P + V + B)/(O + C) ratio (the relative abundance of succinate-producing to succinate-consuming bacteria) correlated positively with myocardial succinate levels, suggesting that the composition of the gut microbial ecosystem in the diabetic state, rather than the absolute abundance of any single species, determines the magnitude of systemic succinate burden. This concept is consistent with recent work demonstrating that oral administration of the succinate-consuming bacterium Odoribacter lanus depletes circulating succinate and improves glycemic control in obese mice (38). Our data therefore extend this therapeutic concept to the cardiac context, supporting the rationale for microbiota-targeted interventions—including probiotics, prebiotics, or dietary modifications—as potential strategies to alleviate diabetic myocardial IR injury.

The disruption of intestinal mucosal integrity—evidenced by the inverse correlation between myocardial succinate levels and tight junction protein (ZO-1 and Occludin) expression—provides a conduit for gut-derived succinate to enter the systemic circulation and reach distant organs. Previous research has shown that diabetes compromises intestinal mucosal integrity, with ZO-1 and occludin serving as essential biomarkers of tight junction integrity (39, 40). Indeed, antibiotic-mediated depletion of the gut microbiota effectively reversed succinate accumulation across all compartments (intestine, cecal contents, serum, and myocardium). Additionally, fecal microbiota transplantation from diabetic donors restored the succinate burden in PGF recipients (**Figure 4A**), establishing a causal role of the gut microbiota in systemic and cardiac succinate overload. The metabolic communication between the gut and the heart is an emerging area of research in the field of cardiovascular science. Our research results indicate that the succinate produced by the gut microbiota plays an important role as a medium in this connection.

Within the myocardium, macrophages constitute the predominant immune cell population and serve as pivotal regulators of post-injury cardiac repair through coordinated necrotic cell clearance, angiogenic stimulation, inflammatory containment, and extracellular matrix remodeling (41). The balance between pro-inflammatory M1 and anti-inflammatory M2 macrophages critically influences infarct size, ventricular remodeling, and functional recovery (42, 43). Notably, macrophage infiltration is markedly elevated in diabetic cardiac tissue, with a pronounced predominance of M1-like macrophages and elevated levels of associated proinflammatory mediators (44). Modulating macrophage polarization towards the anti-inflammatory M2 phenotype and inducing a phenotypic shift from proinflammatory M1 to M2 macrophages represent promising therapeutic strategies to mitigate myocardial IR injury in diabetic contexts. Our study demonstrates that diabetic hearts exhibit a pronounced M1 macrophage predominance, consistent with prior reports, and that succinate directly amplifies this M1 bias.

Succinate, a critical regulator of macrophage-mediated immune responses in metabolic disorders, has attracted considerable interest as a microbiota-derived signaling metabolite (45, 46). Succinate administration significantly increased M1 infiltration while suppressing M2 markers, thereby further decreasing the M2/M1 ratio and exacerbating myocardial IR injury. The critical role of macrophages was confirmed by macrophage depletion experiments, which abrogated succinate-exacerbated IR injury. These *in vivo* findings were corroborated by *in vitro* conditioned medium experiments demonstrating that succinate-stimulated macrophages secrete factors that directly induce cardiomyocyte apoptosis under simulated diabetic conditions. Moreover, loss-of-function experiments conducted with *Sucnr1*^−/−^ mice and Sucnr1-silenced macrophages demonstrated that the SUCNR1 receptor is essential for succinate-induced macrophage polarization.

Mechanistically, our data demonstrate that succinate signals through its cognate receptor SUCNR1 to activate the PI3K/AKT–HIF-1α pathway, a signaling axis previously implicated in macrophage polarization in other contexts (32). Pharmacological inhibition of PI3K or HIF-1α completely abolished succinate-induced M1 polarization *in vitro*, and genetic ablation of *Sucnr1* protected both cultured macrophages and *Sucnr1*^−/−^ mice from succinate-induced inflammatory responses and myocardial injury (**Figure 7**, **Figure 8**). The findings demonstrate that SUCNR1 is essential for succinate-driven polarization of cardiac macrophages, offering insight into how a metabolite from the gut impacts cardiac immune responses under diabetic conditions.

Several limitations of this study should be acknowledged. First, our *in vivo* genetic experiments utilized global *Sucnr1*^−/−^ mice. While our *in vitro* data and macrophage depletion experiments support a macrophage-intrinsic role for SUCNR1, we cannot fully exclude contributions from SUCNR1 expressed on other cell types, such as endothelial cells or cardiomyocytes. Future studies employing macrophage-specific conditional Sucnr1 knockout mice will be necessary to establish the cell-autonomous role of macrophage SUCNR1. Second, we did not include an HFD-only control group to isolate the effects of diet-induced obesity independent of STZ-induced hyperglycemia. Although the HFD+STZ model is widely accepted for modeling T2D, some studies have reported modest succinate elevations following prolonged HFD feeding alone (47). The systemic succinate accumulation observed in our model likely reflects the combined effects of HFD+STZ; the independent contribution of HFD alone remains to be systematically examined. Third, the *in vivo* contribution of the PI3K/HIF-1α pathway was validated genetically through *Sucnr1* ablation but not pharmacologically, as systemic inhibition of PI3K or HIF-1α would exert confounding effects on cardiomyocyte survival. Therefore, the current evidence for this pathway derives primarily from our *in vitro* pharmacological inhibition and genetic knockdown experiments, highlighting the need for future studies using macrophage-specific *Hif1a* knockout approaches.

## 5 Conclusion

Collectively, these findings definitively identify the gut microbiota as the primary source of elevated cardiac succinate levels in individuals with diabetes. Furthermore, we demonstrate that succinate exacerbates myocardial IR injury by promoting SUCNR1-dependent macrophage polarization via activation of the PI3K/HIF-1α signaling axis. These results position microbiota-derived succinate as a promising therapeutic target for alleviating diabetic cardiovascular complications.

## Data Availability

The raw data supporting the conclusions of this article will be made available by the authors, without undue reservation.

## Supplementary material

Supplementary Figure 1 Protein expression levels of occludin and ZO−1 in intestine tissue of diabetic and nondiabetic mice. (n = 3). Data are expressed as mean ± standard error of the mean. ^*^P < 0.05, ^**^P < 0.01, NoDIA: nodiabetes, DIA:diabetes.

Supplementary Figure 2 Gut microbiota diversity is reduced in diabetic mice. **(A–C)** Alpha diversity indices in diabetic and nondiabetic mice: **(A)** Chao1 index, **(B)** Shannon index, and **(C)** Simpson index (n = 9). **(D)** PCoA of beta diversity based on Bray-Curtis distance in diabetic and nondiabetic mice (n = 9). Data are expressed as mean ± standard error of the mean. ^*^P < 0.05, ^**^P < 0.01, ^***^P < 0.001, ^****^P < 0.0001. NoDIA: nodiabetes, DIA:diabetes, PCoA: Principal coordinates analysis.

Supplementary Figure 3 Changes in gut microbiota diversity following antibiotic depletion and fecal microbiota transplantation in diabetic mice. **(A–C)** Alpha diversity indices in diabetic and ABX-treated diabetic (DIA+ABX) mice: **(A)** Chao1 index, **(B)** Shannon index, and **(C)** Simpson index (n = 9). **(D)** PCoA of beta diversity based on Bray-Curtis distance in ABX-treated and FMT recipient mice (n = 9). Data are expressed as mean ± standard error of the mean. ^*^ P < 0.05, ^**^P < 0.01, ^****^P < 0.0001. NoDIA: nodiabetes, DIA:diabetes, ABX: antibiotic mixture, FMT: Faecal microbiota transplantation.

Supplementary Figure 4 Dose-dependent upregulation of M1 marker gene expression by succinate *in vitro*. RAW 264.7 macrophages were treated with increasing concentrations of succinate (0, 0.1, 0.5, 1, and 5 mM) for 24 h. mRNA expression levels of the M1 polarization marker (*Tnf, Il1, Il6, and Nos2*) were determined by RT-qPCR (n = 6). Data are expressed as mean ± standard error of the mean. ^****^P < 0.0001.

Supplementary Figure 5 Body weight and blood glucose levels after HFD and STZ administration. **(A)** Body weight of *Suncr1*^−/−^ and wild-type control mice after HFD+STZ treatment. **(B)** Fasting blood glucose levels measured prior to IR surgery. (n = 9). DIA: diabetes; HFD: high-fat diet; STZ, streptozotocin.
