# Supplementary material for: Gut microbiota-induced elevation of succinate exacerbates diabetic myocardial ischemia/reperfusion injury by promoting macrophage polarization
Source: Front Immunol. 2026 Jun 10;17:1749185. doi: 10.3389/fimmu.2026.1749185 (PMC13290513; doi:10.3389/fimmu.2026.1749185)
Supplement: Supplementary file 1 [file Table1.docx]

**Supplementary Table 1**. Specific primer sequencesused for each gene.

| **Gene** | **Forward** **primer (5’ to 3’)** | **Reverse primer (5’ to 3’)** |
| --- | --- | --- |
| *Il10* | AGGGTCTGGGCCATAGAACT | GCAACCCAAGTAACCCTTAAAG |
| *Tnf* | TGGGACAGTGACCTGGACTGT | TTCGGAAAGCCCATTTGAGT |
| *Il1b* | GGGGCGTCCTTCATATGTGT | GGCAGCTCCTGTCTTGTAGG |
| *Nos2* | AGGTACTCAGCGTGCTCCAC | GCACCGAAGATATCTTCATG |
| *Il6* | TAGTCCTTCCTACCCCAATTTCC | TTGGTCCTTAGCCACTCCTTC |
| *Tjp1* | TTTTTGACAGGGGGAGTGG | TGCTGCAGAGGTCAAAGTTCAAG |
| *Ocln* | ATGTCCGGCCGATGCTCTC | TTTGGCTGCTCTTGGGTCTGTAT |
| ***Arg1***  *Gapdh* | CATATCTGCCAAAGACATCGTG  AGGTCGGTGTGAACGGATTTG | GACATCAAAGCTCAGGTGAATC  TGTAGACCATGTAGTTGAGGTCA |
