# Supplementary figures and images for: Gut microbiota-induced elevation of succinate exacerbates diabetic myocardial ischemia/reperfusion injury by promoting macrophage polarization
Source: Front Immunol. 2026 Jun 10;17:1749185. doi: 10.3389/fimmu.2026.1749185 (PMC13290513; doi:10.3389/fimmu.2026.1749185)

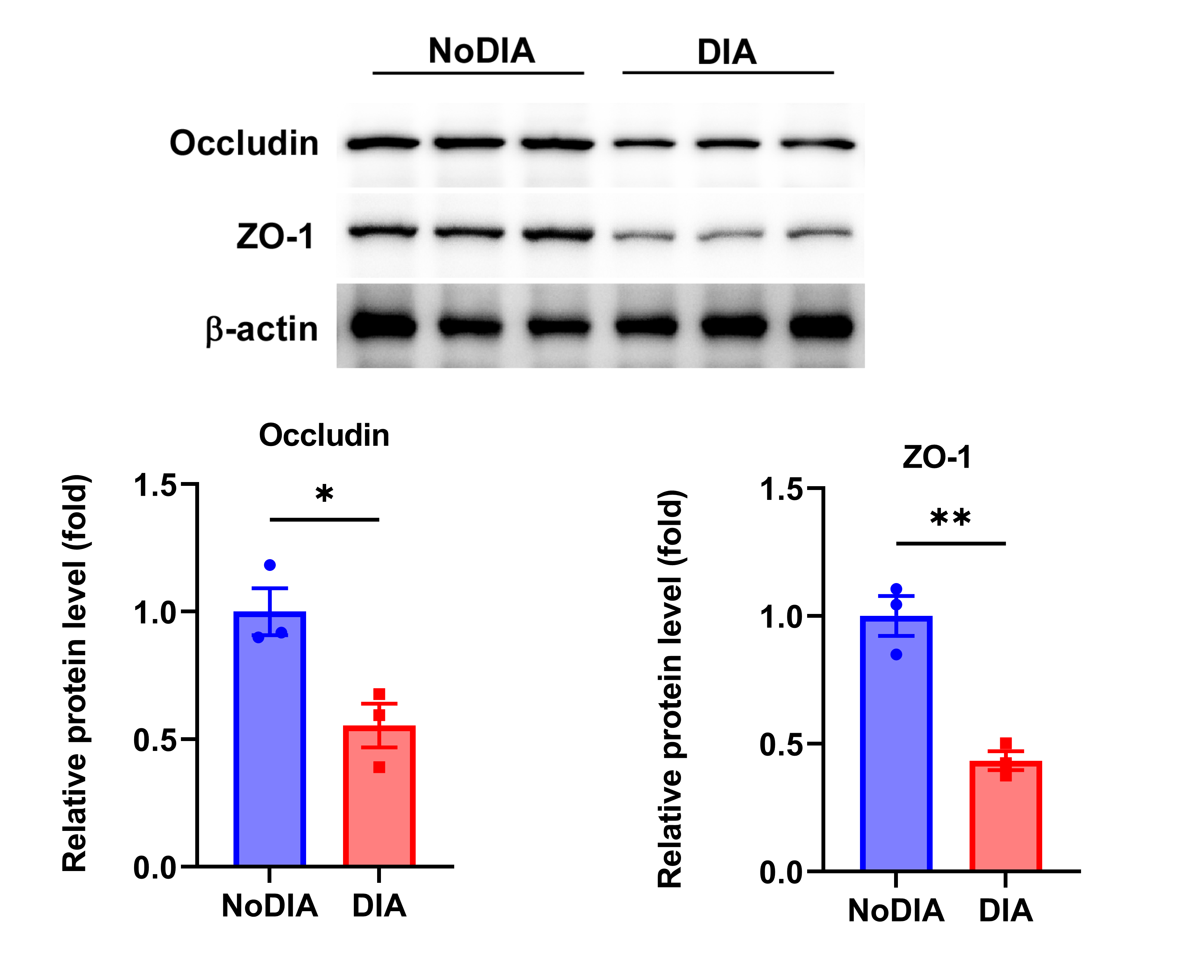

Supplement: Supplementary Figure 1 — Protein expression levels of occludin and ZO−1 in intestine tissue of diabetic and nondiabetic mice. (n = 3). Data are expressed as mean ± standard error of the mean. *P < 0.05, **P < 0.01, NoDIA: nodiabetes, DIA:diabetes. [file Image1.tif]

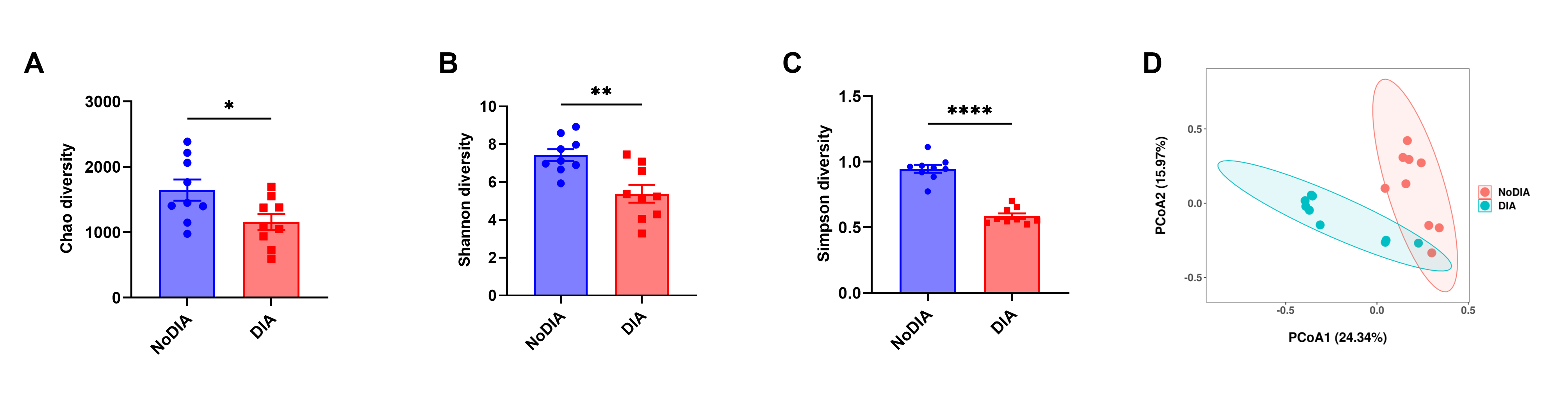

Supplement: Supplementary Figure 2 — Gut microbiota diversity is reduced in diabetic mice. (A–C) Alpha diversity indices in diabetic and nondiabetic mice: (A) Chao1 index, (B) Shannon index, and (C) Simpson index (n = 9). (D) PCoA of beta diversity based on Bray-Curtis distance in diabetic and nondiabetic mice (n = 9). Data are expressed as mean ± standard error of the mean. *P < 0.05, **P < 0.01, ***P < 0.001, ****P < 0.0001. NoDIA: nodiabetes, DIA:diabetes, PCoA: Principal coordinates analysis. [file Image2.tif]

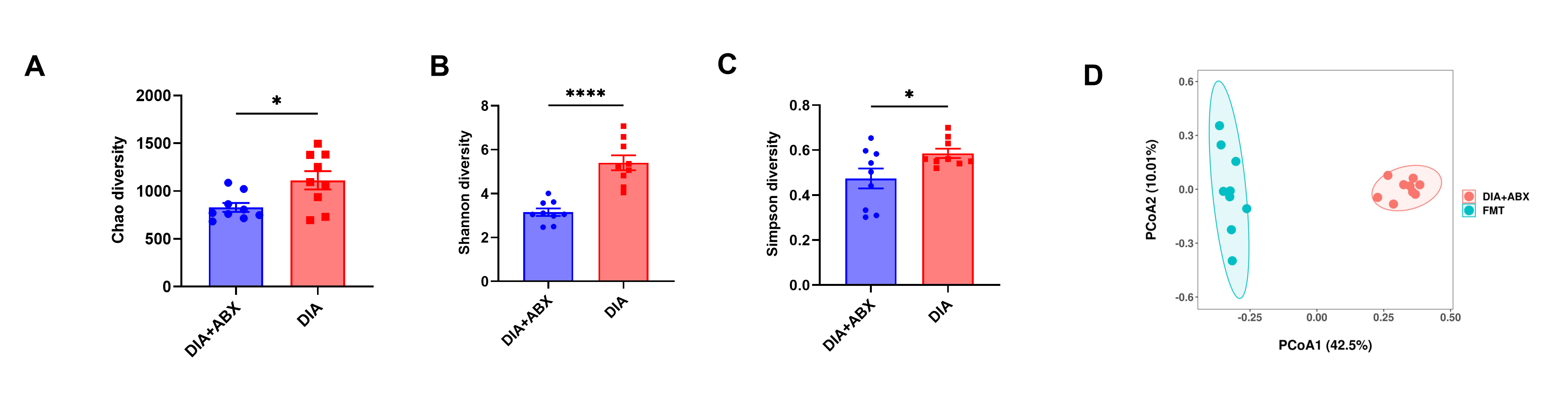

Supplement: Supplementary Figure 3 — Changes in gut microbiota diversity following antibiotic depletion and fecal microbiota transplantation in diabetic mice. (A–C) Alpha diversity indices in diabetic and ABX-treated diabetic (DIA+ABX) mice: (A) Chao1 index, (B) Shannon index, and (C) Simpson index (n = 9). (D) PCoA of beta diversity based on Bray-Curtis distance in ABX-treated and FMT recipient mice (n = 9). Data are expressed as mean ± standard error of the mean. * P < 0.05, **P < 0.01, ****P < 0.0001. NoDIA: nodiabetes, DIA:diabetes, ABX: antibiotic mixture, FMT: Faecal microbiota transplantation. [file Image3.tif]

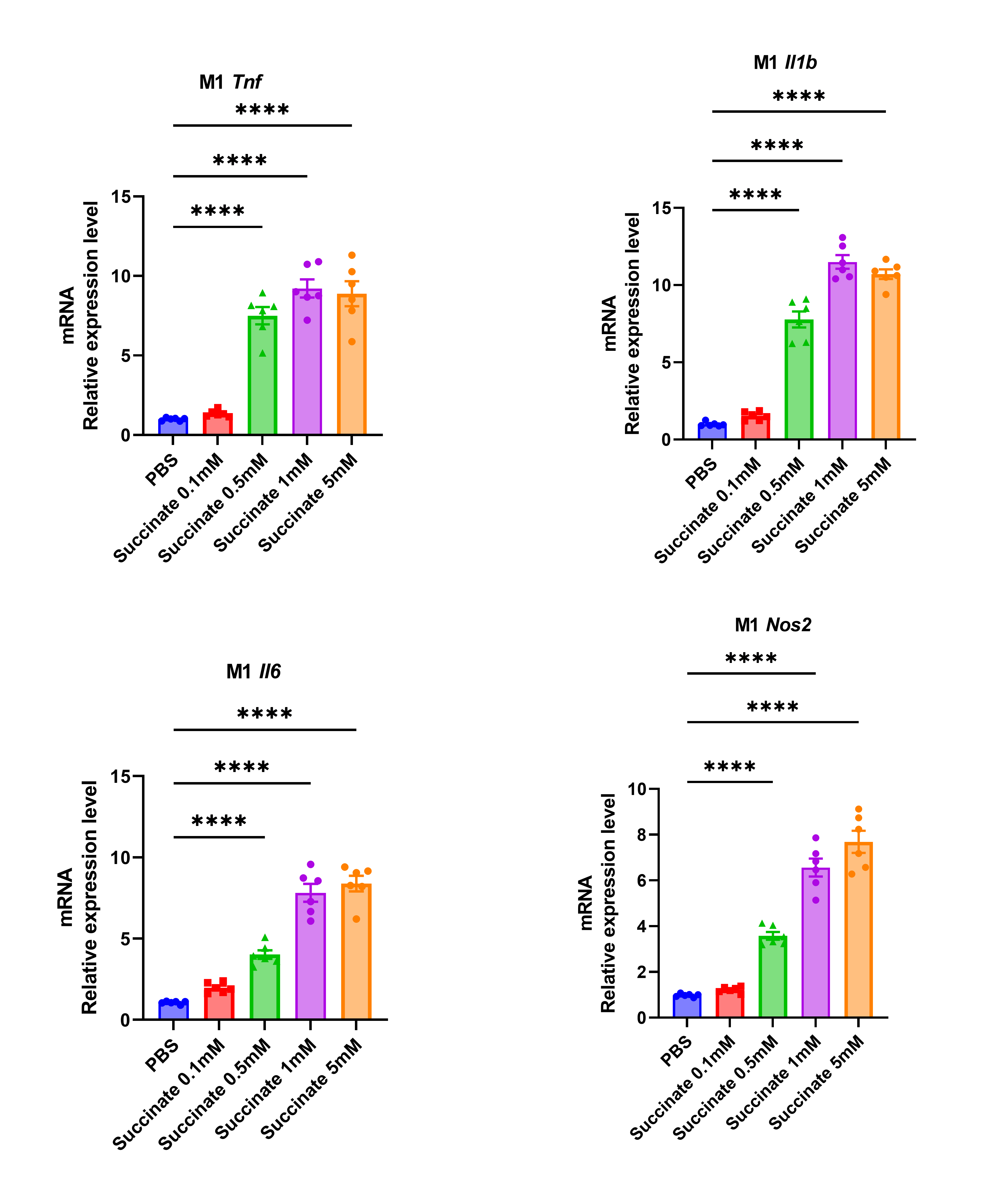

Supplement: Supplementary Figure 4 — Dose-dependent upregulation of M1 marker gene expression by succinate in vitro. RAW 264.7 macrophages were treated with increasing concentrations of succinate (0, 0.1, 0.5, 1, and 5 mM) for 24 h. mRNA expression levels of the M1 polarization marker (Tnf, Il1, Il6, and Nos2) were determined by RT-qPCR (n = 6). Data are expressed as mean ± standard error of the mean. ****P < 0.0001. [file Image4.tif]

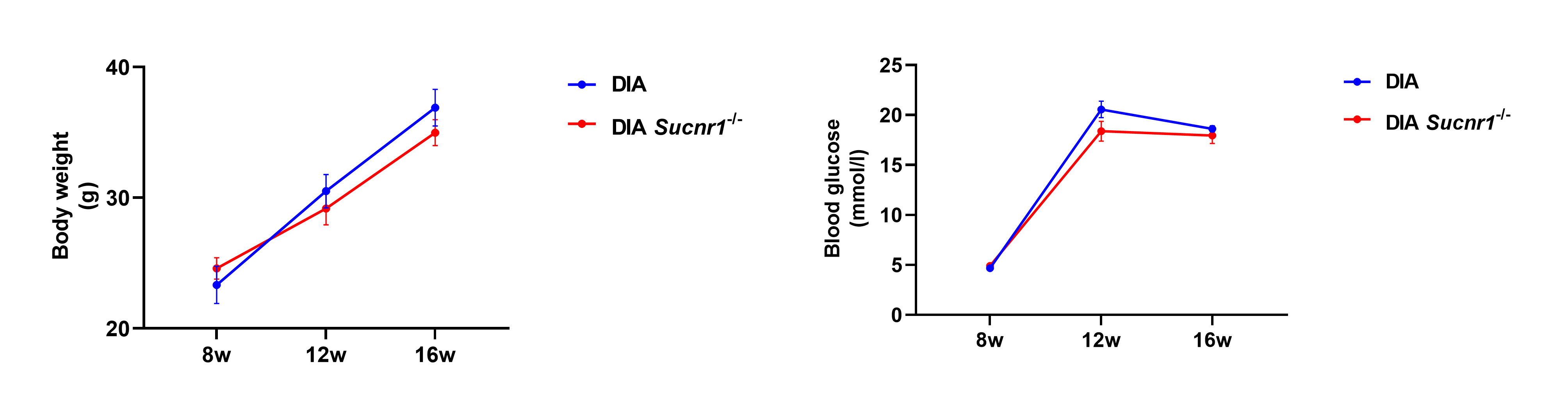

Supplement: Supplementary Figure 5 — Body weight and blood glucose levels after HFD and STZ administration. (A) Body weight of Suncr1−/− and wild-type control mice after HFD+STZ treatment. (B) Fasting blood glucose levels measured prior to IR surgery. (n = 9). DIA: diabetes; HFD: high-fat diet; STZ, streptozotocin. [file Image5.tif]
